# Twenty Years of Cytoreductive Surgery for Advanced Endometrial Carcinoma: A Single-Center Retrospective Cohort Study

**DOI:** 10.3390/cancers18101617

**Published:** 2026-05-16

**Authors:** Britt Kilkens, Eva Maria Roes, Ingrid Boere, Jan-Willem Mens, Heleen van Beekhuizen

**Affiliations:** 1 Department of Gynecologic Oncology, Erasmus MC Cancer Institute, 3015 GD Rotterdam, The Netherlands; 2 Department of Medical Oncology, Erasmus MC Cancer Institute, 3015 GD Rotterdam, The Netherlands; 3 Department of Radiotherapy, Erasmus MC Cancer Institute, 3015 GD Rotterdam, The Netherlands

**Keywords:** endometrial carcinoma, advanced stage, cytoreductive surgery, treatment strategies, overall survival

## Abstract

Objectives: Endometrial carcinoma (EC), the most common gynecological malignancy, is associated with unfavorable survival in advanced stages. Treatment strategies now include cytoreductive surgery (CRS) and (neo)adjuvant chemotherapy, but survival rates remain limited. This study evaluates overall survival (OS) and surgical outcomes, including outcomes of CRS and surgical complications, over a 20-year period at the Erasmus MC. Methods: This retrospective cohort study includes women diagnosed with FIGO stage III or IV EC between 2000 and 2020 who received treatment at the Erasmus MC. Data were collected from the Netherlands Comprehensive Cancer Organization and supplemented by medical record reviews. Statistical analyses were conducted to evaluate differences in OS based on FIGO stage, histological type, molecular characteristics, CRS outcome, and type of CRS. Results: A total of 188 patients were included, with a median age of 66 years. Most patients received surgery and additional chemotherapy and radiotherapy. A total of 64 patients (59.3%) underwent primary CRS, and 44 patients (40.7%) underwent interval CRS. Patients with complete CRS had a significant survival advantage over patients with optimal and incomplete CRS (HR 0.56; 95% CI 0.33–0.96, *p* = 0.036). Comparison between primary and interval CRS revealed no significant difference in OS (HR 1.42; 95% CI 0.82–2.44, *p* = 0.207). Surgical complications occurred in 33.1% of patients, with infections most common. Two patients died from severe complications. Conclusions: This study highlights the predominant role of surgery in the management of advanced EC. Complete CRS is often achievable and offers significant survival advantage. However, approximately one-third of patients experience surgical complications.

## Simple Summary

Endometrial cancer is the most common gynecological cancer, but patients with advanced disease still face poor survival. Treatment usually involves extensive surgery to remove as much tumor as possible, combined with chemotherapy and sometimes radiotherapy. In this study, we analyzed survival and surgical outcomes of patients with advanced endometrial cancer treated over a 20-year period at a tertiary referral center. We found that complete removal of visible tumor during surgery was associated with improved survival, confirming its importance as a key treatment goal. However, about one in three patients experienced surgical complications, and overall survival remained limited. These findings underline the central role of surgery but also the need for careful patient selection and improved treatment strategies. Future advances, particularly in personalized and molecular-based therapies, are needed to improve outcomes for this challenging group of patients.

## 1. Introduction

Endometrial carcinoma (EC) is the most prevalent gynecological malignancy globally [1]. Worldwide, it ranks as the sixth most common type of cancer among females, with 420.368 new cases and 97.723 deaths reported annually [2]. Typically, EC is diagnosed at early stages because symptoms such as postmenopausal vaginal bleeding occur early on [3]. However, approximately 15% of cases present at advanced stages, classified as FIGO (International Federation of Gynecology and Obstetrics) stage III or IV, indicating disease spread outside the uterus, with regional spread or more distant dissemination [4,5]. Common sites of advanced disease involvement include pelvic or para-aortic lymph nodes, the bladder or rectum, omentum, lungs, or liver [6,7]. The 5-year survival rate sharply declines as the disease is more advanced. The 5-year survival rate is 96% for localized disease, compared with 22% for distant disease [8].

Historically, radiotherapy predominantly played a role as adjuvant therapy for advanced EC to prevent local recurrence [9]. However, the current standard for advanced EC often involves cytoreductive surgery (CRS) and (neo)adjuvant chemotherapy with paclitaxel and carboplatin. This paradigm is widely recognized in the treatment of ovarian cancer and is being extrapolated to advanced EC due to comparable metastatic patterns and similar histology [10,11,12,13,14,15]. Despite advancements in the treatment of advanced EC, survival rates continue to be unsatisfactory. Given the unfavorable prognosis, there is a pressing need to explore avenues for improving the complex management of patients with advanced disease [16].

The primary objective of this study is to evaluate overall survival (OS) in patients with advanced EC treated at a tertiary referral center. The secondary objective is to assess surgical outcomes, including the outcomes and type of CRS and surgical complications.

## 2. Materials and Methods

### 2.1. Inclusion Criteria

This retrospective cohort study included all women aged >18 years with histologically confirmed FIGO stage III or IV EC who received any part of their treatment at the Erasmus MC Cancer Institute (Rotterdam, the Netherlands) between January 2000 and January 2020. The Erasmus MC is a tertiary referral center for gynecologic oncology. For patients diagnosed before 2009, FIGO 1988 staging was used, whereas for those diagnosed in 2009 or later, FIGO 2009 criteria were applied.

### 2.2. Data Collection

Data were obtained from the Netherlands Comprehensive Cancer. Collected variables included demographic and disease characteristics (age at diagnosis, histology, FIGO stage, and molecular profile where available), treatment details (treatment intent, timing and type of surgery, outcome CRS, adjuvant or neoadjuvant chemotherapy, radiotherapy, and complications), and follow-up data (vital status and date of death). Missing items and data regarding molecular characteristics were collected by retrospectively reviewing electronic patient files.

Between 2011 and 2020, immunohistochemistry (IHC) for mismatch repair (MMR) proteins and p53, and DNA polymerase-epsilon (POLE) sequencing when available, were performed by a clinical molecular pathologist at Erasmus MC. MMR IHC was performed routinely from 2018 onward, p53 IHC from 2015, and POLE testing only incidentally. Molecular data were included when available in the records.

### 2.3. Outcomes of Interest

The primary outcome of interest is OS, and the primary outcome measure is the adjusted HR for death. Secondary outcomes of interest are surgical complications and the outcome of CRS. Survival is calculated from the incidence date to the end of the follow-up period or death, censoring patients who were alive on the date of the last follow-up (1 January 2023). For the OS analysis, death regardless of the cause is considered an event. Surgical complications were defined as intraoperative or postoperative events occurring within 30 days after surgery.

### 2.4. Surgical Definitions and Criteria

The outcome of CRS was classified as complete (no macroscopically visible residual disease), optimal (residual disease < 1 cm), or incomplete (residual disease ≥ 1 cm). Primary CRS was performed when preoperative evaluation suggested that no or ≤1 cm residual disease could be achieved, and the patient’s condition permitted extensive surgery. Interval CRS followed neoadjuvant chemotherapy when initial imaging or clinical assessment indicated that complete or optimal CRS was unlikely or the patient was unfit for primary surgery.

### 2.5. Statistical Analysis

Survival data were analyzed using IBM SPSS Statistics e version 28.01 (IBM Corp., Armonk, NY, USA). OS is presented in Kaplan–Meier curves. Differences in survival are assessed using Log-Rank tests. Survival data were analyzed by FIGO stage, histological type, molecular characteristics, outcome of CRS, and type of CRS. The Cox proportional hazards model was applied to assess associations between OS and clinical variables. Factors with significant associations in univariable analysis were entered into a multivariable Cox regression model to adjust for potential confounders, including FIGO stage, histological type, and age at diagnosis. All statistical tests were two-sided, and *p* < 0.05 was considered statistically significant.

## 3. Results

### 3.1. Study Population

A total of 188 patients were diagnosed with advanced EC between January 2000 and January 2020. The study population characteristics are shown in Table 1. The median age at primary diagnosis was 66 years, ranging from 29 to 90 years. A total of 84 (44.7%) patients were diagnosed with FIGO stage III EC, while 104 (55.3%) patients were diagnosed with FIGO stage IV EC. The histological type was endometrioid in 95 patients (50.6%), serous in 39 patients (20.7%) and carcinosarcoma in 39 patients (20.7%). The histological type for 15 patients (8.0%) was classified as ‘Other’. Among the endometrioid histological types, 27 cases (28.4%) were differentiation grade one (G1; ≤5% non-squamous solid growth), 24 cases (25.3%) were differentiation grade two (G2; 6–50% solid growth), 43 cases (45.3%) were differentiation grade three (G3; >50% solid growth), and the differentiation grade was unknown for one case (1.0%). Between 2011 and 2020, classification in molecular subtypes was performed on 112 patients with advanced EC at the Erasmus MC (Table 1), using IHC of MMR proteins and p53. POLE testing was not yet conducted in this period of time. Among these, 19 (17.0%) were MMR deficient (MMRd), regardless of p53 status, 61 (54.4%) were MMR proficient (MMRp) or MMR unknown with p53 mutant (p53mt) staining, 19 patients (17.0%) were MMRp or MMR unknown with p53 wild-type (p53wt) staining, and 13 (11.6%) were MMRp with unknown p53 status.

### 3.2. Treatment Strategies

Treatment characteristics of the study population are shown in Table 1. The majority of patients was initially treated with curative intent (*n* = 145, 77.1%), all of whom underwent surgery. The remaining patients (*n* = 43, 22.9%) were treated with palliative intent from the start of treatment. In the curative setting, 37 patients underwent a hysterectomy with bilateral salpingo-oophorectomy, and 108 patients underwent CRS. Seven patients underwent an exploratory laparotomy at first and later underwent a second interval CRS, with six patients ultimately achieving complete CRS and one achieving optimal CRS. In total, of the CRS procedures, 15 surgeries were incomplete (13.9%), 16 were optimal (14.8%), and 77 were complete (71.3%). A total of 64 patients underwent primary CRS (59.3%), and 44 patients underwent interval CRS (40.7%) after neoadjuvant chemotherapy.

Table 2 displays the combination of treatment modalities administered to patients. Most patients underwent surgery and received adjuvant treatment with both chemotherapy and radiotherapy. In a curative setting, radiotherapy was administered to 60 out of 74 patients (81.1%) with stage III and to 20 out of 71 patients (28.2%) with stage IV. Among the 60 stage III patients who received radiotherapy, three (5%) received brachytherapy alone, 28 (46.7%) received external beam radiotherapy (EBRT), and 29 (48.3%) received both EBRT and brachytherapy. Among the 20 stage IV patients who received radiotherapy, six (30%) underwent EBRT, 12 (60%) received brachytherapy, and two (10%) received both EBRT and brachytherapy. Palliative radiotherapy was used to irradiate the uterus to reduce vaginal bleeding or irradiate distant metastases in 11 patients (33.3%). In the curative setting, 44 out of 74 patients (59.5%) with stage III and 61 out of 71 patients (85.9%) with stage IV received chemotherapy. Of the total 105 patients who received chemotherapy, 44 patients (41.9%) received neoadjuvant chemotherapy before CRS. The majority received six cycles (with a maximum of 12) of carboplatin and paclitaxel. In cases of interval CRS, typically three cycles were administered before CRS and three cycles after. A total of 23 patients received chemotherapy as palliative treatment.

### 3.3. Survival

The median OS for the whole study population was 22 months (95%CI 16.9–27.1). Cox univariable analysis of the cohort revealed that age at diagnosis, FIGO stage, and histological type significantly affected OS.

**Figure A1:**
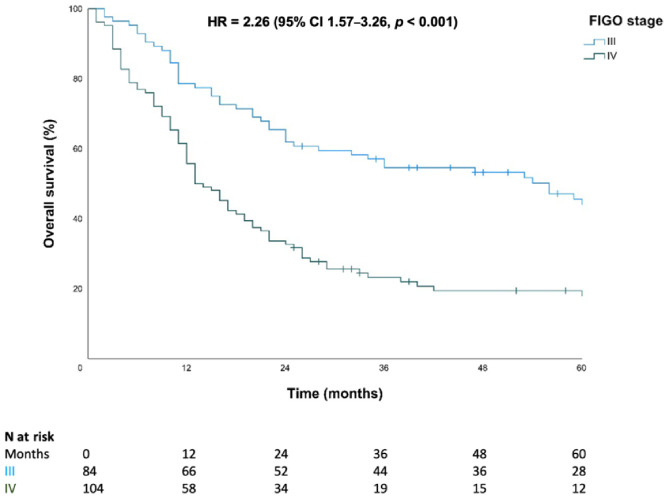
Kaplan–Meier plot comparing overall survival between patients with stage III and stage IV advanced EC. Short vertical lines indicate censored observations (patients alive at last follow-up).

The 5-year survival rate for patients with FIGO stage III was 44%. For patients with FIGO stage IV, the 5-year survival rate was 17.9%. The median OS for patients with FIGO stage III and IV was 56 (95%CI 34.0–78.0) and 13 (95%CI 9.4–16.6) months, respectively. The OS of patients with FIGO stage III or IV was significantly different (*p* < 0.001) (Figure A1). Adjusted for histological type and age at diagnosis, FIGO stage IV was associated with a significant disadvantage in survival over FIGO stage III with a HR of 2.26 (95% CI 1.57–3.26, *p* < 0.001).

**Figure A2:**
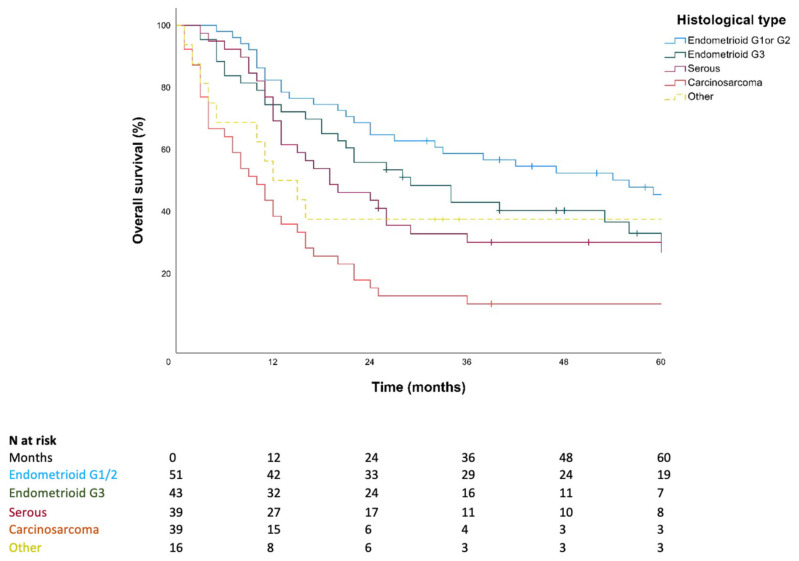
Kaplan–Meier plot comparing overall survival between patients with different histological types of advanced EC: endometrioid G1/G2, endometrioid G3, serous, carcinosarcoma, and other. Short vertical lines indicate censored observations (patients alive at last follow-up).

Regarding histological type, there were significant differences in OS between endometrioid G1/2 EC and serous EC (*p* = 0.012) and endometrioid G1/2 EC and carcinosarcomas (*p* < 0.001). The difference between endometrioid G1/2 and endometrioid G3 EC was not significant (*p* = 0.170) (Figure A2). After controlling for FIGO stage and age at diagnosis, there was no significant difference between endometrioid G1/2 EC and endometrioid G3 EC (HR 0.72; 95%CI 0.43–1.21, *p* = 0.213) and between endometrioid G1/2 EC and serous EC (HR 1.04; 95%CI 0.62–1.74, *p* = 0.897). On multivariable analysis, only carcinosarcoma is associated with significantly worse OS compared to all other histological types (Table A1).

**Figure A3:**
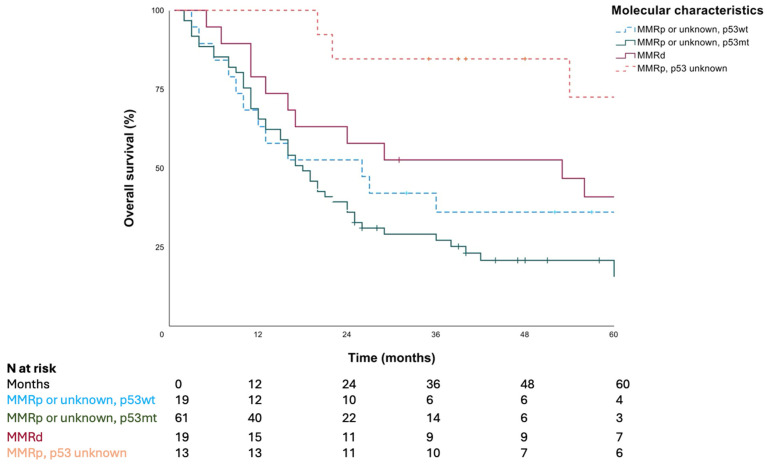
Kaplan–Meier plot comparing overall survival between patients with advanced EC with different molecular characteristics: MMRp/unknown p53wt, MMRp/unknown p53mt, MMRd and MMRp p53 unknown. Short vertical lines indicate censored observations (patients alive at last follow-up).

As for molecular characteristics, there was a significant difference in OS between the MMRp/unknown p53mt group and the MMRd group (*p* = 0.035) (Figure A3). However, on multivariable analysis, no significant survival benefit of MMRd compared to MMRp/unknown p53mt was observed (HR 0.62; 95% CI 0.32–1.23, *p* = 0.171). There were no other relevant differences in OS regarding molecular characteristics.

**Figure 1:**
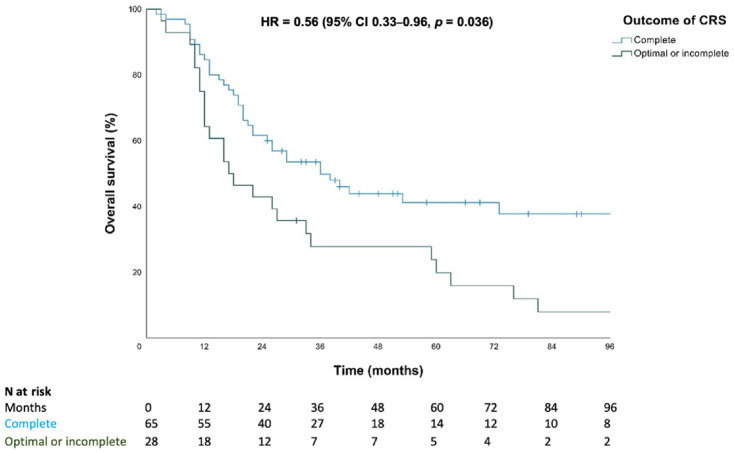
Kaplan–Meier plot comparing overall survival between patients with complete CRS versus optimal or incomplete CRS in advanced EC. Short vertical lines indicate censored observations (patients alive at last follow-up).

Figure 1 shows the survival curves for patients who underwent CRS with (neo)adjuvant therapy by the outcome of CRS. After 5 years, 41.4% of the patients with complete CRS were alive, compared to 19.8% of the patients with optimal or incomplete CRS. For patients with optimal or incomplete CRS, the median OS was 17 months (95% CI 7.7–26.3), while it was 36 months (95% CI 20.7–51.3) for patients with complete CRS. The difference in OS between complete and optimal or incomplete CRS was statistically significant (*p* = 0.005). Adjusted for FIGO stage, histological type, and age at diagnosis, among patients who underwent CRS, patients with complete CRS had a survival advantage over optimal and incomplete CRS with a HR of 0.56 (95% CI 0.33–0.96, *p* = 0.036).

**Figure A4:**
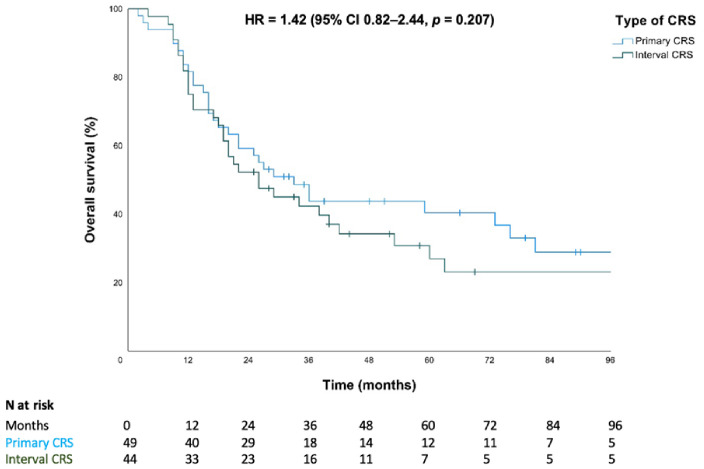
Kaplan–Meier plot comparing overall survival between patients with advanced EC who underwent primary CRS and interval CRS. Short vertical lines indicate censored observations (patients alive at last follow-up).

Comparison between primary and interval CRS showed no significant difference in OS (HR 1.42; 95% CI 0.82–2.44, *p* = 0.207) (Figure A4).

### 3.4. Surgical Complications

A total of 48 patients (33.1%) experienced complications because of surgery (Table 3). The most common complication was an infection (*n* = 27), primarily affecting the urinary tract (*n* = 11), surgical wound (*n* = 4) or lungs (*n* = 4). Five patients developed sepsis, leading to the death of one patient. Other frequent complications were damage to an organ (*n* = 19) or functional issues (*n* = 12). Organ damage most commonly occurred in the lower urinary tract (*n* = 7), intestines (*n* = 6), or the diaphragm, resulting in a pneumothorax (*n* = 3). Functional complications included ileus (*n* = 8), severe constipation (*n* = 2), or a dysfunctional bladder (*n* = 2). Less common complications included bleeding (*n* = 5), surgery-related thrombosis (*n* = 3), and wound dehiscence (*n* = 3). Two patients experienced a cardiac arrest, one after bleeding and one after hypoxia under anesthesia, resulting in the death of the patient who experienced bleeding. In total, two patients died due to surgical complications. Apart from the two fatal cases, all patients recovered fully.

## 4. Discussion

Our findings underscore the critical role of maximal tumor removal in improving outcomes and provide real-world evidence on long-term survival and perioperative risks in a heterogeneous patient population. While treatment strategies for advanced endometrial cancer have evolved over the past decades, particularly through integration of chemotherapy and radiotherapy, survival gains remain modest, highlighting the ongoing challenges in managing advanced disease. Taken together, our results set the stage for a comparison with the broader literature on CRS outcomes, both in endometrial and ovarian cancer, to contextualize the prognostic value of complete cytoreduction.

From the extensive literature on CRS for advanced OC, it is evident that CRS is effective and the absence of residual disease after surgery correlates with a more favorable survival outcome than the presence of residual tumor [14,15]. However, studies investigating the role of CRS in advanced EC are more limited, often retrospective, and involve relatively small numbers of patients [10,12].

In an analysis of 55 EC and 110 OC patients, Landrum et al. [16] compared outcomes of CRS for advanced EC and OC, controlling for age and residual disease. They confirmed that a residual tumor size of less than one centimeter independently predicts improved OS in both EC and OC patients (*p* = 0.01). Other retrospective observational studies also demonstrate a survival benefit, especially with complete CRS, in advanced EC following (neo)adjuvant chemotherapy [17,18].

More recently, Albright et al. [19] conducted a meta-analysis including 34 studies involving patients with stage III as well as stage IV EC (*n* = 1329). Based on reported hazard ratios, the meta-analysis showed that optimal and incomplete CRS were associated with worse OS (optimal: HR 2.57; 95%CI 2.13–3.10; I2 = 1%; incomplete: HR 2.62; 95%CI 2.20–3.11; I2 = 15%) compared to complete CRS. Sensitivity analyses limited to high quality studies demonstrated consistent results.

These findings align with our own results, emphasizing the importance of maximal cytoreduction in advanced EC.

In addition, evidence from OC studies suggest that chemotherapy administered before surgery may be considered for patients who are not suitable candidates for primary CRS. Randomized controlled trials in OC have demonstrated that similar survival outcomes are achieved with interval CRS compared to primary CRS in FIGO stage III or IV OC [20,21,22].

In a retrospective cohort study using a national cancer database, Tobias et al. [23] analyzed 4890 patients with stage IV EC to compare the survival outcomes between patients receiving primary chemotherapy versus primary surgery. Their findings indicated that neoadjuvant chemotherapy improved survival in the first three to eight months but increased mortality afterward.

In a retrospective analysis by Eto et al. [24], primary CRS was compared with interval CRS in patients (*n* = 426) with stage IVB EC. OS was similar between the two groups among patients who ultimately underwent surgery after neoadjuvant chemotherapy.

Our findings are consistent with these observations, showing no significant difference in OS between primary and interval CRS. This may partly reflect that complete cytoreduction was achieved in many patients across both groups. Importantly, interval CRS is often performed in patients with more extensive disease or who are initially considered less fit for surgery. The comparable survival outcomes observed therefore support the feasibility and effectiveness of interval CRS in appropriately selected patients, highlighting that achieving maximal cytoreduction is a key determinant of outcome regardless of the timing of surgery.

In the context of curative treatment, adjuvant treatment for advanced EC can include radiotherapy, chemotherapy, or a combination. Traditionally, in Europe, radiotherapy has been the standard adjuvant treatment following surgery. However, newer treatment paradigms have integrated chemotherapy into the regimen [9,25].

The PORTEC-3 trial [25] compared adjuvant chemoradiotherapy (CTRT) to radiotherapy alone in 660 women with high-risk EC. The trial initially found that adjuvant CTRT, compared to RT alone, did not significantly improve 5-year OS in high-risk EC (HR 0.76; 95% CI 0.54–1.06, *p* = 0.11), although it did improve 5-year failure-free survival (HR 0.71; 95% CI 0.53–0.95, *p* = 0.022). However, post hoc analyses revealed significant OS benefits after adjustment for stratification factors: 81.4% vs. 76.1% (HR 0.70; 95% CI 0.51–0.97, *p* = 0.034) overall, and for stage III disease specifically, 78.5% vs. 68.5% (HR 0.63; 95% CI 0.41–0.99, *p* = 0.043). For serous EC, CTRT significantly improved 5-year OS to 71.4% vs. 52.8% (HR 0.48; 95% CI 0.24–0.96, *p* = 0.037) [26]. Molecular subgroup analysis further highlighted improved 5-year recurrence-free survival for p53-abnormal tumors treated with CTRT (59% vs. 36%, *p* = 0.019) [27]. These findings suggest that this treatment schedule may be recommended, especially for women with stage III, serous EC, or p53-abnormal tumors, albeit it at the cost of increased toxicity. These nuanced results emphasize the prognostic value of molecular classification and the importance of individualized treatment decisions based on the specific characteristics and stage of the disease. Although our findings did not mirror the significant prognostic associations regarding molecular characteristics, the limitations of sample size and incomplete molecular data in our study may account for this discrepancy.

The GOG-122 trial [28] compared whole abdominal irradiation with doxorubicin/cisplatin chemotherapy in stage III and IV patients (*n* = 396) who underwent optimal CRS. The findings from this study confirmed the role of adjuvant chemotherapy in treating lymph node positive EC, after results showed significant survival differences between the two groups in favor of the chemotherapy arm (stage adjusted death HR 0.68; 95%CI 0.52–0.89, *p* < 0.01). Stage-adjusted predictions indicated that at 60 months, 50% of patients receiving chemotherapy were alive and disease-free, compared to 38% of those receiving whole abdominal irradiation.

Building on these findings, the GOG-258 trial [29] explored whether adding EBRT to post surgery chemotherapy could improve outcomes for patients with locally advanced EC. The results indicated that adding radiation to chemotherapy may not significantly improve relapse-free survival in patients with FIGO stage III or IVA EC (HR, 0.90; 90% CI, 0.74 to 1.10, *p* = 0.20).

Although adjuvant radiotherapy remains an option for patients with advanced EC, adjuvant chemotherapy seems to improve survival. In our study, there were different combinations of treatment regimens for stage III and stage IV, underscoring the challenge in assessing the exact role of chemotherapy alone.

Although our retrospective dataset contained detailed counts of adverse events rather than patient-level complication grading, formal comparative analysis between primary and interval CRS groups was not feasible due to the absence of patient-level complication grading and the inability to account for multiple events per patient. Nevertheless, our overall complication rate of 33% lies within the range reported in previous series on advanced EC, in which complication rates between 25% and 40% have been described [7,30,31]. In earlier reports by Ayhan et al. [30] and Bristow et al. [7], severe (grade III–V) surgical morbidity occurred in roughly one quarter of patients, mainly due to infections, bowel injury, and hemorrhage, patterns comparable to those observed in our cohort. Importantly, the frequency of complications did not appear to differ between primary and interval CRS in those studies, suggesting that the degree of cytoreduction rather than the timing of surgery may determine perioperative risk. The heterogeneity in surgical approach, patient selection, and reporting standards across institutions limits direct comparison, but our results corroborate the consistent finding that complete cytoreduction, while achievable in selected patients, carries significant morbidity that demands highly experienced multidisciplinary care.

### 4.1. Future Research

Complete CRS is recognized as a favorable prognostic factor, not only in advanced OC but also in advanced EC. The optimization of surgical techniques holds promise as a potential avenue for improving survival outcomes by increasing the likelihood of achieving complete CRS. The use of surgical innovations such as the PlasmaJet, which is already being investigated in OC, may contribute to achieving this goal [32]. Therefore, exploring surgical techniques to achieve complete CRS is essential to improve outcomes.

After extensive research on the use of hyperthermic intraperitoneal chemotherapy (HIPEC) in advanced OC, HIPEC has become an established part of its treatment for selected patients [33]. However, the role of HIPEC in the management of advanced EC is limitedly explored, and its use is not yet integrated into treatment strategies, despite similarities in metastatic patterns and serous histology between advanced OC and advanced EC. While some small studies suggest that HIPEC may have a positive impact on survival outcomes, stronger evidence from larger prospective trials is needed before HIPEC can be integrated into the treatment of advanced EC [34].

Over the past two decades, adjuvant therapy selection has primarily been based on clinico-pathologic characteristics. However, molecular characteristics with prognostic value are increasingly influencing treatment strategies and are now routinely determined depending on availability [9]. Recently, immunotherapy, particularly checkpoint inhibitors (anti-PD-1, anti-PD-L1), has been shown to substantially improve the prognosis of patients with advanced or recurrent MMRd EC. Several trials demonstrated significant improvements in progression-free survival and OS in patients with advanced or recurrent MMRd EC following the addition of checkpoint inhibitors such as dostarlimab, pembrolizumab, atezolizumab or durvalumab, to chemotherapy. The role of immunotherapy in advanced or recurrent MMRp EC is less clear, but studies with immune checkpoint inhibitors have also shown improved clinical outcomes [35,36,37,38]. In the adjuvant setting the benefit of immunotherapy is not yet crystallized, the ENGOT EN11/Keynote B21 study could not demonstrate a benefit in terms of disease-free survival in the whole population of stage I/II high risk endometrial cancer [38]. There was a benefit in the MMRd population. However, other studies need to be awaited. The RAINBO studies investigate different adjuvant treatment strategies based on the molecular profile of EC. These include immunotherapy (durvalumab) for MMRd EC, olaparib (PARP inhibitor) for p53 mutant EC, treatment de-escalation for POLE- mutated tumors, and hormonal treatment strategies for the no specific molecular profile (NSMP) and estrogen receptor positive tumors. In some cases, the presence of specific mutations can be used to select patients with a lower chance of recurrence, making adjuvant chemotherapy unnecessary. POLE- mutated tumors are currently the best example where adjuvant chemotherapy can be safely omitted [39].

Upcoming trends involve a transition towards molecular-based risk classification and personalized treatment modalities and combinations. These novel treatments offer promise in altering survival outcomes. Therefore, molecular classification is necessary at the beginning to make the best treatment schedule. Future research should focus on the exploration of improved treatments for advanced EC based on molecular characteristics and how to combine this with chemotherapy and radiotherapy [40].

### 4.2. Strengths and Limitations

However, this study has several limitations. The sample size of 188 patients provides valuable insight but limits the statistical power, particularly for subgroup analyses such as comparisons between histological types or molecular subtypes. With a larger cohort, smaller but clinically meaningful differences might have reached statistical significance.

The retrospective design introduces several potential sources of bias, including selection and information bias, as outcomes are derived from existing patient records. Differences in data completeness and documentation over time may have affected the accuracy of certain variables, especially complications and molecular subtypes. To minimize this, we included only patients treated at the Erasmus MC, ensuring access to comprehensive medical records and allowing detailed validation of both clinical and pathological data. This approach reduces the risk of incomplete or inconsistent data inherent to multicenter registry studies, although it also limits broader generalizability.

As a result, our findings primarily reflect the experience of a single tertiary referral center, where surgical decision-making and perioperative care are delivered by specialized teams. Outcomes may therefore differ in institutions with varying expertise or resource availability. Nevertheless, this single-center design enhances internal consistency and provides an in-depth view of long-term clinical practice within a highly specialized setting.

The long inclusion period of this study (2000–2020) spanned substantial changes in treatment strategies, surgical techniques, and diagnostic approaches, including the gradual introduction of molecular classification. These changes may have influenced treatment decisions, surgical outcomes, and survival, adding variability that is inherent to retrospective studies. Differences in chemotherapy protocols, radiotherapy techniques, and the timing of CRS (primary vs. interval) may also have introduced confounding effects that could not be fully adjusted for, despite multivariable analysis. This heterogeneity reflects everyday clinical practice but should be taken into account when interpreting the results. Molecular subtyping was not consistently available throughout the study period because these techniques were implemented gradually, which limited the possibility of performing detailed stratified molecular analyses.

Moreover, potential selection bias must be taken into account. Patients who underwent complete CRS generally represent those with better performance status, lower disease burden, or more favorable tumor distribution, which may have contributed to the observed survival advantage in this group. This inherent limitation of retrospective surgical studies underscores that the association between complete CRS and improved survival, while consistent with prior evidence, should be interpreted with caution.

The FIGO staging was revised in 2009, with the newly adopted staging being independent of positive peritoneal cytology and only including cases with uterine serosa and/or adnexal involvement for stage IIIA. Although our included patients were staged before 2009 as well as after 2009, only two patients were staged as IIIA based on positive peritoneal cytology before 2009. Also, the WHO classification system for endometrial carcinomas was updated twice, in 2003 and 2014. These updates introduced refinements in diagnostic criteria, placing greater importance on the use of molecular techniques and IHC, enabling more accurate subtype classification. This means that cases classified in our study as endometrioid, serous, or carcinosarcoma might now, under the updated criteria, be reclassified as a different subtype. However, no major shifts occurred in distinguishing endometrioid carcinomas, serous carcinomas and carcinosarcomas based on morphological features, so the impact on our results is expected to be minimal [41].

Furthermore, our study population consisted of patients with advanced EC, defined as FIGO stage III and IV. In FIGO stage III, the disease is limited to locoregional or lymphogenic metastases, while in FIGO stage IV, intra-abdominal or distant metastases may be present. This heterogeneity in metastatic sites makes it challenging to draw conclusions for the entire group, highlighting the importance of a personalized approach based on patient specific clinico-pathologic characteristics.

This study has notable strengths. Firstly, it provides unique insights into the treatment of advanced EC and associated outcomes, particularly within a major institution. The extensive and detailed retrospective cohort offers valuable analysis of treatment strategies, surgical complications, and survival outcomes that are not often available in the literature. The long study period and comprehensive clinical documentation enabled an evaluation of changes in surgical management and patient outcomes over time, offering a realistic view of evolving clinical practice. All procedures were conducted within the same tertiary referral center, ensuring consistency in surgical approach and multidisciplinary decision-making, which strengthens the internal validity of the findings.

## 5. Conclusions

In this study, we evaluated OS and surgical outcomes in patients with advanced EC. OS remained limited but was significantly associated with the completeness of cytoreductive surgery. Patients who achieved complete CRS showed improved survival compared with those with residual disease. However, surgical treatment was associated with a considerable rate of postoperative complications. Given the retrospective design and relatively small sample size, our findings should be interpreted cautiously and mainly provide insight into outcomes within a tertiary referral center. The role of molecular classification and personalized treatment of EC will be increasingly important and should therefore be the main focus of future research.

## Figures and Tables

**Table 1:** Study population and treatment characteristics.

Characteristic	N (%)
Total	188 (100)
Age	
Median (range)	66 (2990)
FIGO stage	
IIIA	23 (12.2)
IIIB	10 (5.3)
IIIC1	27 (14.4)
IIIC2	24 (12.8)
IVA	13 (6.9)
IVB	91 (48.4)
Histological type	
Endometrioid	95 (50.6)
Serous	39 (20.7)
Carcinosarcoma	39 (20.7)
Other	15 (8.0)
Adenocarcinoma *	3 (1.6)
Clear cell	4 (2.1)
Undifferentiated/dedifferentiated	5 (2.7)
Neuroendocrine	2 (1.1)
Mixed	1 (0.5)
Differentiation grade of endometrioid EC	
G1	27 (28.4)
G2	24 (25.3)
G3	43 (45.3)
Unknown	1 (1.0)
Molecular characteristics	
MMRd	19 (17.0)
MMRp/unknown, p53mt	61 (54.4)
MMRp/unknown, p53wt	19 (17.0)
MMRp, p53 unknown	13 (11.6)
Type of surgery	
Hysterectomy with bilateral salpingo-oophorectomy	37 (25.0)
Cytoreductive surgery	108 (73.0)
Palliative setting	3 (2.0)
Type of CRS	
Primary	64 (59.3)
Interval	44 (40.7)
Outcome of CRS	
Complete	77 (71.3)
Optimal	16 (14.8)
Incomplete	15 (13.9)
Chemotherapy	
(Neo)adjuvant	105 (82.0)
Palliative	23 (18.0)
Radiotherapy	
Adjuvant	80 (72.9)
Palliative	11 (12.1)

FIGO = International Federation of Gynecology and Obstetrics; MMRd = mismatch repair deficient; MMRp = mismatch repair proficient; p53mt = p53 mutant; p53wt = p53 wild-type; * not further specified. Percentages for CRS type and outcome are based on the 108 patients who underwent CRS.

**Table 2:** Initial treatment according to FIGO stage.

FIGO Stage	S + R	S + C	S + C + R	NAC + S + C	NAC + S + C + R	C	S	N	O
IIIA	10	1	7	0	0	0	1	0	4
IIIB	4	0	1	0	1	0	1	1	2
IIIC	8	3	19	3	6	2	1	1	8
IVA	0	2	2	3	1	0	1	1	3
IVB	0	11	7	19	6	12	5	11	20
Total	22	17	36	25	14	14	9	14	37

S = surgery; R = radiotherapy; C = adjuvant chemotherapy; NAC = neoadjuvant chemotherapy before surgery; N = no active oncologic treatment; O = other combinations. Columns represent the actual treatment combinations administered. Palliative cases are included across the categories according to the treatment received.

**Table 3:** Surgical complications *.

Complication Category	Subtype	N (%)
Any complication	–	48 (33.1)
Infections	Total	27 (18.6)
	Urinary tract	11 (7.6)
	Surgical wound	4 (2.8)
	Pulmonary	4 (2.8)
	Sepsis	5 (3.4)
Organ damage	Total	19 (13.1)
	Lower urinary tract	7 (4.8)
	Intestinal	6 (4.1)
	Diaphragm/pneumothorax	3 (2.1)
Functional complications	Total	12 (8.3)
	Ileus	8 (5.5)
	Severe constipation	2 (1.4)
	Bladder dysfunction	2 (1.4)
Bleeding	–	5 (3.4)
Surgery-related thrombosis	–	3 (2.1)
Wound dehiscence	–	3 (2.1)
Cardiac arrest	–	2 (1.4)
Deaths due to surgical complications	–	2 (1.4)

* Complications include intraoperative and postoperative events occurring within 30 days after surgery. Patients may have experienced more than one complication.

## Data Availability

The data supporting the findings of this study are available from the corresponding author upon reasonable request. The data are not publicly available due to privacy restrictions.

## Abbreviations

The following abbreviations are used in this manuscript:

EC	Endometrial carcinoma
OS	Overall survival
FIGO	International Federation of Gynecology and Obstetrics
CRS	Cytoreductive surgery
IHC	Immunohistochemistry
MMR	Mismatch repair
POLE	DNA polymerase-epsilon
EBRT	External beam radiotherapy
RT	Radiotherapy
CTRT	Chemoradiotherapy
HIPEC	Hyperthermic intraperitoneal chemotherapy

## Appendix A

### Appendix A.3

**Table A1:** Survival outcomes by histological type.

	Endometrioid G1/G2	Endometrioid G3	Serous	Carcinosarcoma	Other
5-year survival	45.4%	28.9%	26.7%	10.3%	37.5%
Median OS in months	56	29	19	10	12
HR	1	1.39 (*p* = 0.213)	1.44 (*p* = 0.180)	3.65 (*p* < 0.001)	1.66 (*p* = 0.172)
HR	0.72 (*p* = 0.213)	1	1.04 (*p* = 0.897)	2.63 (*p* < 0.001)	1.20 (*p* = 0.633)
HR	0.70 (*p* = 0.180)	0.97 (*p* = 0.897)	1	2.54 (*p* < 0.001)	1.16 (*p* = 0.705)
HR	0.27 (*p* < 0.001)	0.38 (*p* < 0.001)	0.40 (*p* < 0.001)	1	0.46 (*p* = 0.033)
HR	0.60 (*p* = 0.172)	0.84 (*p* = 0.633)	0.87 (*p* = 0.705)	2.20 (*p* = 0.033)	1
